# Aqua­bis­(3-chloro­benzoato-κ*O*)bis­(*N*,*N*-di­ethyl­nicotinamide-κ*N*)copper(II)

**DOI:** 10.1107/S1600536813018989

**Published:** 2013-07-17

**Authors:** Nihat Bozkurt, Tuncay Tunç, Nagihan Çaylak Delibaş, Hacali Necefoğlu, Tuncer Hökelek

**Affiliations:** a Department of Chemistry, Kafkas University, 36100 Kars, Turkey; b Aksaray University, Science Education Department, 68100, Aksaray, Turkey; c Department of Physics, Sakarya University, 54187 Esentepe, Sakarya, Turkey; d Department of Physics, Hacettepe University, 06800 Beytepe, Ankara, Turkey

## Abstract

The title compound, [Cu(C_7_H_4_ClO_2_)_2_(C_10_H_14_N_2_O)_2_(H_2_O)], has twofold symmetry with the Cu^II^ cation and the O atom of the coordinating water mol­ecule located on the axis. The Cu^II^ cation is coordinated by two carboxyl­ate O atoms of chloro­benzoate (CB) anions, two N atoms of *N*,*N*-di­ethyl­nicotinamide (DENA) ligands and one water mol­ecule in a distorted N_2_O_3_ square-pyramidal geometry. The benzene and pyridine rings are oriented at a dihedral angle of 82.51 (6)°. In the anionic ligand, the carboxyl­ate group is twisted away from the attached benzene ring by 12.85 (11)°. In the crystal, O—H⋯O hydrogen bonds between the coordinating water mol­ecule and the carboxyl group link the complex mol­ecules into supra­molecular chains running along the *c-*axis direction.

## Related literature

For literature on niacin, see: Krishnamachari (1974 ▶). For information on the nicotinic acid derivative *N*,*N*-di­ethyl­nicotinamide, see: Bigoli *et al.* (1972 ▶). For related structures, see: Hökelek *et al.* (1996 ▶, 2009*a* ▶,*b* ▶); Hökelek & Necefoğlu (1998 ▶, 2007 ▶); Necefoğlu *et al.* (2011*a* ▶,*b* ▶,*c* ▶). For bond-length data, see: Allen *et al.* (1987 ▶).
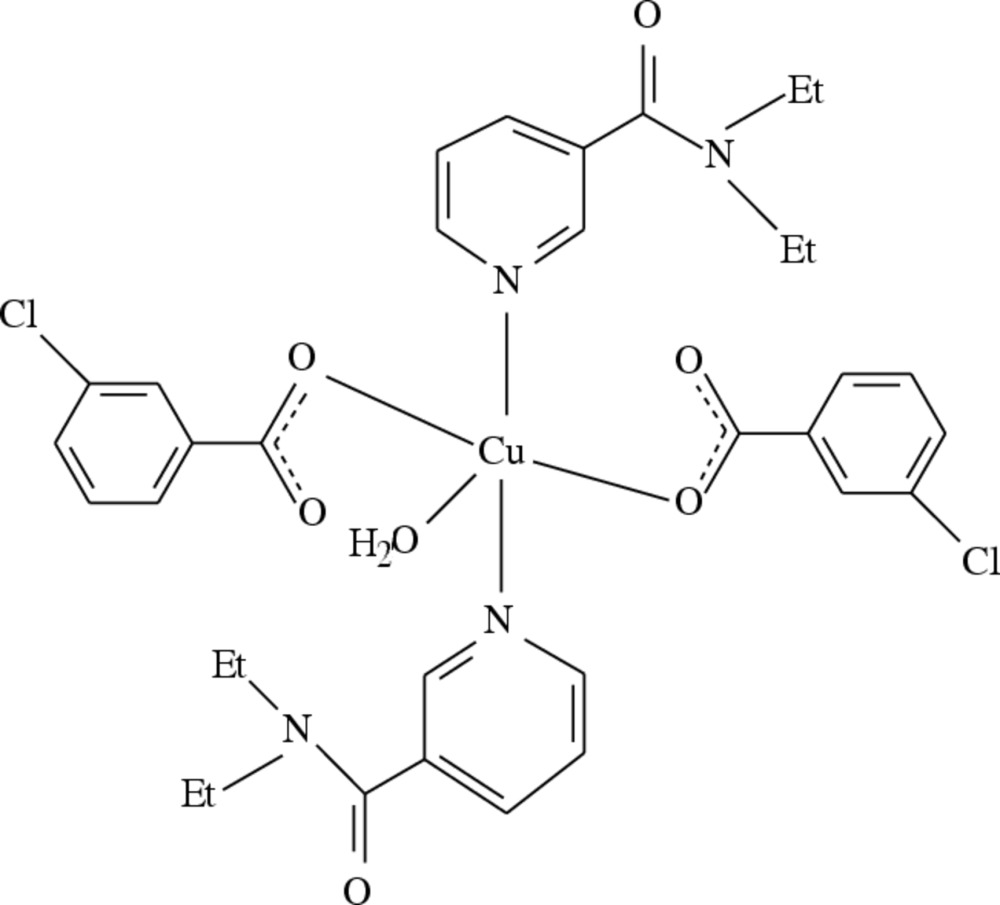


## Experimental

### 

#### Crystal data

[Cu(C_7_H_4_ClO_2_)_2_(C_10_H_14_N_2_O)_2_(H_2_O)]
*M* *_r_* = 749.13
Orthorhombic, 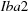

*a* = 15.9185 (9) Å
*b* = 19.2366 (11) Å
*c* = 11.5535 (7) Å
*V* = 3537.9 (4) Å^3^
*Z* = 4
Mo *K*α radiation
μ = 0.82 mm^−1^
*T* = 296 K
0.35 × 0.20 × 0.15 mm

#### Data collection

Bruker SMART BREEZE CCD diffractometer
Absorption correction: multi-scan (*SADABS*; Bruker, 2012 ▶) *T*_min_ = 0.821, *T*_max_ = 0.884
75834 measured reflections
6232 independent reflections
5189 reflections with *I* > 2σ(*I*)
*R*_int_ = 0.048

#### Refinement

*R*[*F* ^2^ > 2σ(*F* ^2^)] = 0.032
*wR*(*F* ^2^) = 0.086
*S* = 1.06
6232 reflections
224 parameters
1 restraint
H atoms treated by a mixture of independent and constrained refinement
Δρ_max_ = 0.25 e Å^−3^
Δρ_min_ = −0.39 e Å^−3^
Absolute structure: Flack (1983 ▶), with no Friedel pairs measured
Flack parameter: 0.027 (10)

### 

Data collection: *APEX2* (Bruker, 2012 ▶); cell refinement: *SAINT* (Bruker, 2012 ▶); data reduction: *SAINT*; program(s) used to solve structure: *SHELXS97* (Sheldrick, 2008 ▶); program(s) used to refine structure: *SHELXL97* (Sheldrick, 2008 ▶); molecular graphics: *ORTEP-3* for Windows (Farrugia, 2012 ▶); software used to prepare material for publication: *WinGX* (Farrugia, 2012 ▶) and *PLATON* (Spek, 2009 ▶).

## Supplementary Material

Crystal structure: contains datablock(s) I, global. DOI: 10.1107/S1600536813018989/xu5719sup1.cif

Structure factors: contains datablock(s) I. DOI: 10.1107/S1600536813018989/xu5719Isup2.hkl

Additional supplementary materials:  crystallographic information; 3D view; checkCIF report

## Figures and Tables

**Table 1:** Selected bond lengths (Å)

Cu1—N1	2.0294 (12)
Cu1—O1	1.9337 (10)
Cu1—O4	2.238 (2)

**Table 2:** Hydrogen-bond geometry (Å, °)

*D*—H⋯*A*	*D*—H	H⋯*A*	*D*⋯*A*	*D*—H⋯*A*
O4—H41⋯O2^i^	0.79 (4)	1.95 (3)	2.7367 (17)	171 (4)

Symmetry code: (i) 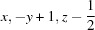.

**Fig. 1.**
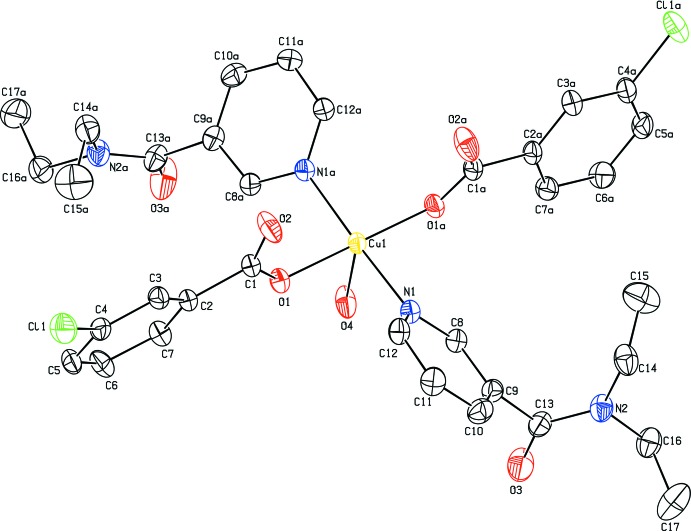
The molecular structure of the title molecule with the atom-numbering scheme. Displacement ellipsoids are drawn at the 50% probability level [symmetry code: (a) 2 - x, 1 - y, z]. Hydrogen atoms have been omitted for clarity.

## supplementary crystallographic information

### Comment

As a part of our ongoing investigations of transition metal complexes of nicotinamide (NA), one form of niacin (Krishnamachari, 1974), and/or the nicotinic acid derivative *N*,*N*-diethylnicotinamide (DENA), an important respiratory stimulant (Bigoli *et al.*, 1972), the title compound was synthesized and its crystal structure is reported herein.

The asymmetric unit of the title mononuclear Cu^II^ complex, (Fig. 1), contains one-half molecule, the Cu^II^ cation is located on a twofold rotation axis and is coordinated by carboxylate O atoms of two chlorobenzoate (CB) anions, N atoms of two *N*,*N*-diethylnicotinamide (DENA) ligands and by one water molecule, located on a twofold rotation axis, all ligands coordinating in a monodentate manner. The crystal structures of similar complexes of Cu^II^, Co^II^, Ni^II^, Mn^II^ and Zn^II^ cations, [Cu(C_7_H_5_O_2_)_2_(C_10_H_14_N_2_O)_2_] (Hökelek *et al.*, 1996); [Cu(C_9_H_9_O_2_)_2_(C_10_H_14_N_2_O)_2_(H_2_O)_2_] (Necefoğlu *et al.*, 2011*a*); [Cu(C_7_H_4_FO_2_)_2_(C_10_H_14_N_2_O)_2_(H_2_O)_2_] (Necefoğlu *et al.*, 2011*b*); [Co(C_6_H_6_N_2_O)_2_(C_7_H_4_NO_4_)_2_(H_2_O)_2_] (Hökelek & Necefoğlu, 1998); [Co(C_9_H_9_O_2_)_2_(C_10_H_14_N_2_O)_2_(H_2_O)_2_] (Necefoğlu *et al.*, 2011*c*); [Ni(C_7_H_4_ClO_2_)_2_(C_6_H_6_N_2_O)_2_(H_2_O)_2_] (Hökelek *et al.*, 2009*a*); [Mn(C_9_H_10_NO_2_)_2_(H_2_O)_4_].2H_2_O (Hökelek & Necefoğlu, 2007) and [Zn(C_7_H_4_BrO_2_)_2_(C_6_H_6_N_2_O)_2_(H_2_O)_2_] (Hökelek *et al.*, 2009*b*) have also been reported. In the first copper(II) complex mentioned above the two benzoate ions coordinate to the Cu^II^ atom as bidentate ligands, while in the other structures all the ligands coordinate in a monodentate manner.

In the title complex, the four symmetry related O and N atoms (O1, O1a and N1, N1a) [symmetry code: (a) - *x*, 1 - *y*, *z*] in the equatorial plane around the Cu^II^ cation form a distorted square-planar arrangement, while the distorted square-pyramidal coordination is completed by the water O atom (O4) in the axial position.

The Cu—O bond lengths are 1.9346 (11) Å (for benzoate oxygen) and 2.238 (2) Å (for water oxygen), and the Cu—N bond length is 2.0303 (14) Å, close to standard values (Allen *et al.*, 1987). The Cu atom is displaced out of the mean-plane of the carboxylate group (O1/C1/O2) by -0.1606 (1) Å. The dihedral angle between the planar carboxylate group and the adjacent benzene ring *A* (C2—C7) is 12.85 (11)°. The benzene *A* (C2—C7) and the pyridine *B* (N1/C8—C12) rings are oriented at a dihedral angle of 82.51 (6)°.

In the crystal, strong O—H···O hydrogen bonds (Table 2) link the water hydrogens to the carboxylate oxygens into infinite chains along the c-axis.

### Experimental

The title compound was prepared by the reaction of CuSO_4_.5H_2_O (1.25 g, 5 mmol) in H_2_O (100 ml) and diethylnicotinamide (1.78 g, 10 mmol) in H_2_O (20 ml) with sodium 3-chlorobenzoate (1.79 g, 10 mmol) in H_2_O (100 ml). The mixture was set aside to crystallize at ambient temperature for five days, giving blue single crystals.

### Refinement

Atom H41 (for H_2_O) was located in a difference Fourier map and was refined freely. The C-bound H-atoms were positioned geometrically with C—H = 0.93, 0.97 and 0.96 Å, for aromatic, methylene and methyl H-atoms, respectively, and constrained to ride on their parent atoms, with *U*_iso_(H) = k × *U*_eq_(C), where k = 1.5 for methyl H-atoms and k = 1.2 for all other H-atoms.

### Crystal data

**Table:**

[Cu(C_7_H_4_ClO_2_)_2_(C_10_H_14_N_2_O)_2_(H_2_O)]	*F*(000) = 1556
*M**_r_* = 749.13	*D*_x_ = 1.406 Mg m^−^^3^
Orthorhombic, *I**b**a*2	Mo *K*α radiation, λ = 0.71073 Å
Hall symbol: I 2 -2c	Cell parameters from 9278 reflections
*a* = 15.9185 (9) Å	θ = 2.2–30.7°
*b* = 19.2366 (11) Å	µ = 0.82 mm^−^^1^
*c* = 11.5535 (7) Å	*T* = 296 K
*V* = 3537.9 (4) Å^3^	Block, blue
*Z* = 4	0.35 × 0.20 × 0.15 mm

### Data collection

**Table:**

Bruker SMART BREEZE CCD diffractometer	6232 independent reflections
Radiation source: fine-focus sealed tube	5189 reflections with *I* > 2σ(*I*)
Graphite monochromator	*R*_int_ = 0.048
φ and ω scans	θ_max_ = 32.3°, θ_min_ = 1.7°
Absorption correction: multi-scan (*SADABS*; Bruker, 2012)	*h* = −23→23
*T*_min_ = 0.821, *T*_max_ = 0.884	*k* = −28→28
75834 measured reflections	*l* = −17→16

### Refinement

**Table:**

Refinement on *F*^2^	Secondary atom site location: difference Fourier map
Least-squares matrix: full	Hydrogen site location: inferred from neighbouring sites
*R*[*F*^2^ > 2σ(*F*^2^)] = 0.032	H atoms treated by a mixture of independent and constrained refinement
*wR*(*F*^2^) = 0.086	*w* = 1/[σ^2^(*F*_o_^2^) + (0.049*P*)^2^ + 0.4582*P*] where *P* = (*F*_o_^2^ + 2*F*_c_^2^)/3
*S* = 1.06	(Δ/σ)_max_ = 0.002
6232 reflections	Δρ_max_ = 0.25 e Å^−^^3^
224 parameters	Δρ_min_ = −0.39 e Å^−^^3^
1 restraint	Absolute structure: Flack (1983), with no Friedel pairs measured
Primary atom site location: structure-invariant direct methods	Flack parameter: 0.027 (10)

### Special details

**Table:**

Geometry. All esds (except the esd in the dihedral angle between two l.s. planes) are estimated using the full covariance matrix. The cell esds are taken into account individually in the estimation of esds in distances, angles and torsion angles; correlations between esds in cell parameters are only used when they are defined by crystal symmetry. An approximate (isotropic) treatment of cell esds is used for estimating esds involving l.s. planes.
Refinement. Refinement of F^2^ against ALL reflections. The weighted R-factor wR and goodness of fit S are based on F^2^, conventional R-factors R are based on F, with F set to zero for negative F^2^. The threshold expression of F^2^ > 2sigma(F^2^) is used only for calculating R-factors(gt) etc. and is not relevant to the choice of reflections for refinement. R-factors based on F^2^ are statistically about twice as large as those based on F, and R- factors based on ALL data will be even larger.

### Fractional atomic coordinates and isotropic or equivalent isotropic displacement parameters (Å^2^)

**Table:**

	*x*	*y*	*z*	*U*_iso_*/*U*_eq_	
Cu1	1.0000	0.5000	0.41873 (3)	0.03000 (6)	
Cl1	1.46828 (3)	0.59979 (3)	0.63513 (5)	0.06445 (15)	
O1	1.11786 (6)	0.52434 (6)	0.41852 (13)	0.0393 (2)	
O2	1.14688 (8)	0.52348 (10)	0.60832 (13)	0.0614 (4)	
O3	0.84439 (11)	0.72659 (11)	0.16031 (16)	0.0800 (5)	
O4	1.0000	0.5000	0.22505 (18)	0.0538 (6)	
H41	1.0395 (19)	0.4937 (15)	0.185 (4)	0.083 (10)*	
N1	0.96843 (7)	0.60210 (6)	0.42656 (14)	0.0353 (2)	
N2	0.74305 (10)	0.72799 (9)	0.29566 (14)	0.0469 (3)	
C1	1.16668 (9)	0.52875 (9)	0.50510 (15)	0.0363 (3)	
C2	1.25768 (9)	0.54254 (8)	0.47400 (14)	0.0349 (3)	
C3	1.31396 (9)	0.56202 (9)	0.56019 (15)	0.0391 (3)	
H3	1.2964	0.5673	0.6364	0.047*	
C4	1.39704 (10)	0.57329 (8)	0.52927 (16)	0.0414 (3)	
C5	1.42506 (9)	0.56541 (8)	0.4176 (2)	0.0469 (3)	
H5	1.4812	0.5729	0.3992	0.056*	
C6	1.36773 (12)	0.54600 (10)	0.33236 (19)	0.0514 (4)	
H6	1.3857	0.5403	0.2563	0.062*	
C7	1.28426 (11)	0.53510 (9)	0.36030 (17)	0.0422 (4)	
H7	1.2460	0.5228	0.3030	0.051*	
C8	0.90811 (10)	0.62540 (8)	0.35534 (15)	0.0372 (3)	
H8	0.8802	0.5936	0.3084	0.045*	
C9	0.88569 (10)	0.69476 (8)	0.34881 (14)	0.0398 (3)	
C10	0.92796 (12)	0.74174 (8)	0.4190 (2)	0.0510 (4)	
H10	0.9146	0.7888	0.4161	0.061*	
C11	0.99002 (12)	0.71828 (10)	0.4931 (2)	0.0509 (4)	
H11	1.0188	0.7490	0.5409	0.061*	
C12	1.00827 (10)	0.64755 (10)	0.49440 (18)	0.0412 (3)	
H12	1.0497	0.6314	0.5443	0.049*	
C13	0.82193 (13)	0.71794 (10)	0.26044 (15)	0.0462 (4)	
C14	0.71426 (12)	0.71772 (12)	0.4142 (2)	0.0551 (4)	
H14A	0.6784	0.7563	0.4361	0.066*	
H14B	0.7625	0.7177	0.4656	0.066*	
C15	0.6667 (2)	0.65112 (17)	0.4298 (3)	0.0907 (8)	
H15A	0.6454	0.6486	0.5074	0.136*	
H15B	0.7036	0.6125	0.4159	0.136*	
H15C	0.6208	0.6496	0.3760	0.136*	
C16	0.68061 (14)	0.75374 (11)	0.2115 (2)	0.0546 (5)	
H16A	0.6255	0.7363	0.2325	0.066*	
H16B	0.6943	0.7358	0.1353	0.066*	
C17	0.6778 (2)	0.83180 (13)	0.2067 (3)	0.0864 (9)	
H17A	0.6405	0.8462	0.1461	0.130*	
H17B	0.7332	0.8495	0.1916	0.130*	
H17C	0.6581	0.8496	0.2795	0.130*	

### Atomic displacement parameters (Å^2^)

**Table:**

	*U*^11^	*U*^22^	*U*^33^	*U*^12^	*U*^13^	*U*^23^
Cu1	0.02380 (9)	0.03532 (10)	0.03087 (11)	0.00119 (8)	0.000	0.000
Cl1	0.0404 (2)	0.0777 (3)	0.0752 (4)	−0.0116 (2)	−0.0184 (2)	0.0065 (3)
O1	0.0265 (4)	0.0465 (5)	0.0449 (5)	−0.0019 (4)	−0.0005 (5)	−0.0018 (7)
O2	0.0334 (6)	0.1087 (12)	0.0420 (7)	−0.0084 (7)	0.0062 (5)	0.0074 (8)
O3	0.0700 (10)	0.1258 (14)	0.0442 (8)	0.0380 (10)	0.0141 (8)	0.0253 (10)
O4	0.0329 (9)	0.1020 (18)	0.0265 (10)	0.0133 (9)	0.000	0.000
N1	0.0290 (5)	0.0383 (5)	0.0385 (7)	0.0010 (4)	0.0002 (6)	−0.0028 (6)
N2	0.0421 (7)	0.0617 (8)	0.0369 (7)	0.0086 (6)	−0.0025 (6)	−0.0013 (7)
C1	0.0271 (6)	0.0404 (8)	0.0414 (8)	0.0011 (5)	0.0034 (5)	0.0022 (6)
C2	0.0259 (6)	0.0380 (7)	0.0408 (8)	0.0008 (5)	0.0023 (6)	0.0036 (6)
C3	0.0304 (6)	0.0462 (8)	0.0409 (8)	−0.0006 (6)	−0.0011 (6)	0.0060 (6)
C4	0.0292 (6)	0.0394 (7)	0.0556 (10)	−0.0005 (6)	−0.0050 (6)	0.0073 (7)
C5	0.0283 (6)	0.0440 (7)	0.0683 (10)	−0.0026 (5)	0.0105 (8)	0.0012 (10)
C6	0.0425 (9)	0.0593 (10)	0.0523 (11)	−0.0073 (7)	0.0181 (8)	−0.0060 (8)
C7	0.0353 (7)	0.0488 (9)	0.0426 (9)	−0.0033 (6)	0.0052 (7)	−0.0042 (7)
C8	0.0340 (7)	0.0392 (7)	0.0385 (8)	0.0023 (5)	−0.0017 (6)	−0.0016 (6)
C9	0.0390 (7)	0.0417 (7)	0.0389 (8)	0.0065 (6)	0.0043 (6)	0.0030 (6)
C10	0.0548 (9)	0.0352 (6)	0.0631 (10)	0.0041 (6)	0.0000 (11)	−0.0029 (10)
C11	0.0458 (9)	0.0426 (9)	0.0643 (12)	−0.0027 (7)	−0.0054 (8)	−0.0141 (8)
C12	0.0339 (7)	0.0463 (8)	0.0435 (9)	0.0026 (6)	−0.0029 (6)	−0.0071 (7)
C13	0.0503 (9)	0.0505 (9)	0.0377 (9)	0.0151 (8)	0.0031 (7)	0.0035 (7)
C14	0.0438 (8)	0.0812 (12)	0.0405 (8)	0.0058 (8)	0.0010 (9)	−0.0060 (12)
C15	0.099 (2)	0.0903 (18)	0.0825 (19)	−0.0149 (15)	0.0178 (19)	0.0077 (18)
C16	0.0501 (10)	0.0630 (11)	0.0508 (10)	0.0082 (8)	−0.0108 (8)	−0.0045 (9)
C17	0.094 (2)	0.0615 (13)	0.104 (2)	0.0226 (13)	−0.0383 (17)	−0.0114 (14)

### Geometric parameters (Å, º)

**Table:**

Cu1—N1	2.0294 (12)	C7—H7	0.9300
Cu1—N1^i^	2.0294 (12)	C8—H8	0.9300
Cu1—O1	1.9337 (10)	C9—C8	1.383 (2)
Cu1—O1^i^	1.9337 (10)	C9—C10	1.388 (3)
Cu1—O4	2.238 (2)	C9—C13	1.507 (2)
Cl1—C4	1.7439 (18)	C10—C11	1.383 (3)
O1—C1	1.269 (2)	C10—H10	0.9300
O4—H41	0.79 (4)	C11—H11	0.9300
N1—C8	1.341 (2)	C12—C11	1.391 (3)
N1—C12	1.335 (2)	C12—H12	0.9300
N2—C13	1.334 (2)	C13—O3	1.222 (2)
N2—C14	1.458 (3)	C14—C15	1.499 (4)
N2—C16	1.476 (3)	C14—H14A	0.9700
C1—O2	1.238 (2)	C14—H14B	0.9700
C1—C2	1.516 (2)	C15—H15A	0.9600
C2—C3	1.391 (2)	C15—H15B	0.9600
C2—C7	1.387 (2)	C15—H15C	0.9600
C3—C4	1.387 (2)	C16—C17	1.503 (3)
C3—H3	0.9300	C16—H16A	0.9700
C5—C4	1.373 (3)	C16—H16B	0.9700
C5—C6	1.394 (3)	C17—H17A	0.9600
C5—H5	0.9300	C17—H17B	0.9600
C6—H6	0.9300	C17—H17C	0.9600
C7—C6	1.383 (2)		
			
O1—Cu1—O1^i^	179.86 (9)	C9—C8—H8	118.6
O1—Cu1—O4	89.93 (5)	C8—C9—C10	118.11 (16)
O1^i^—Cu1—O4	89.93 (5)	C8—C9—C13	119.75 (16)
O1—Cu1—N1	90.35 (5)	C10—C9—C13	121.96 (15)
O1^i^—Cu1—N1	89.66 (5)	C9—C10—H10	120.2
O1—Cu1—N1^i^	89.66 (5)	C11—C10—C9	119.69 (15)
O1^i^—Cu1—N1^i^	90.35 (5)	C11—C10—H10	120.2
N1—Cu1—O4	92.55 (5)	C10—C11—C12	118.36 (17)
N1^i^—Cu1—O4	92.55 (5)	C10—C11—H11	120.8
N1^i^—Cu1—N1	174.89 (9)	C12—C11—H11	120.8
C1—O1—Cu1	127.53 (12)	N1—C12—C11	122.34 (17)
Cu1—O4—H41	126 (3)	N1—C12—H12	118.8
C8—N1—Cu1	118.25 (11)	C11—C12—H12	118.8
C12—N1—Cu1	122.85 (12)	O3—C13—N2	122.97 (18)
C12—N1—C8	118.79 (13)	O3—C13—C9	118.98 (17)
C13—N2—C14	124.25 (16)	N2—C13—C9	118.05 (16)
C13—N2—C16	118.78 (16)	N2—C14—C15	112.8 (2)
C14—N2—C16	116.93 (15)	N2—C14—H14A	109.0
O1—C1—C2	114.20 (14)	N2—C14—H14B	109.0
O2—C1—O1	126.72 (15)	C15—C14—H14A	109.0
O2—C1—C2	119.08 (15)	C15—C14—H14B	109.0
C3—C2—C1	119.53 (15)	H14A—C14—H14B	107.8
C7—C2—C1	119.85 (14)	C14—C15—H15A	109.5
C7—C2—C3	120.62 (14)	C14—C15—H15B	109.5
C2—C3—H3	120.9	C14—C15—H15C	109.5
C4—C3—C2	118.15 (16)	H15A—C15—H15B	109.5
C4—C3—H3	120.9	H15A—C15—H15C	109.5
C3—C4—Cl1	119.02 (14)	H15B—C15—H15C	109.5
C5—C4—Cl1	118.68 (12)	N2—C16—C17	112.26 (19)
C5—C4—C3	122.30 (16)	N2—C16—H16A	109.2
C4—C5—C6	118.74 (14)	N2—C16—H16B	109.2
C4—C5—H5	120.6	C17—C16—H16A	109.2
C6—C5—H5	120.6	C17—C16—H16B	109.2
C5—C6—H6	119.8	H16A—C16—H16B	107.9
C7—C6—C5	120.31 (18)	C16—C17—H17A	109.5
C7—C6—H6	119.8	C16—C17—H17B	109.5
C2—C7—H7	120.1	C16—C17—H17C	109.5
C6—C7—C2	119.87 (17)	H17A—C17—H17B	109.5
C6—C7—H7	120.1	H17A—C17—H17C	109.5
N1—C8—C9	122.71 (15)	H17B—C17—H17C	109.5
N1—C8—H8	118.6		
			
O4—Cu1—O1—C1	−173.97 (13)	O1—C1—C2—C7	−13.2 (2)
N1—Cu1—O1—C1	93.48 (14)	O2—C1—C2—C3	−12.1 (2)
N1^i^—Cu1—O1—C1	−81.41 (14)	O2—C1—C2—C7	167.34 (19)
O1—Cu1—N1—C8	133.82 (13)	C1—C2—C3—C4	179.08 (15)
O1^i^—Cu1—N1—C8	−46.04 (13)	C7—C2—C3—C4	−0.4 (2)
O1—Cu1—N1—C12	−42.21 (15)	C1—C2—C7—C6	−178.40 (16)
O1^i^—Cu1—N1—C12	137.94 (15)	C3—C2—C7—C6	1.1 (3)
O4—Cu1—N1—C8	43.87 (12)	C2—C3—C4—Cl1	178.47 (12)
O4—Cu1—N1—C12	−132.15 (14)	C2—C3—C4—C5	−0.4 (2)
Cu1—O1—C1—O2	−6.0 (3)	C6—C5—C4—Cl1	−178.34 (14)
Cu1—O1—C1—C2	174.59 (9)	C6—C5—C4—C3	0.6 (3)
Cu1—N1—C8—C9	−175.86 (13)	C4—C5—C6—C7	0.1 (3)
C12—N1—C8—C9	0.3 (3)	C2—C7—C6—C5	−0.9 (3)
Cu1—N1—C12—C11	175.38 (16)	C10—C9—C8—N1	0.2 (3)
C8—N1—C12—C11	−0.6 (3)	C13—C9—C8—N1	175.42 (16)
C14—N2—C13—O3	179.6 (2)	C8—C9—C10—C11	−0.5 (3)
C14—N2—C13—C9	−0.3 (3)	C13—C9—C10—C11	−175.56 (19)
C16—N2—C13—O3	−2.8 (3)	C8—C9—C13—O3	−79.9 (3)
C16—N2—C13—C9	177.28 (16)	C8—C9—C13—N2	100.0 (2)
C13—N2—C14—C15	−102.9 (3)	C10—C9—C13—O3	95.1 (3)
C16—N2—C14—C15	79.5 (3)	C10—C9—C13—N2	−85.0 (2)
C13—N2—C16—C17	−88.5 (3)	C9—C10—C11—C12	0.2 (3)
C14—N2—C16—C17	89.2 (3)	N1—C12—C11—C10	0.4 (3)
O1—C1—C2—C3	167.34 (15)		

Symmetry code: (i) −*x*+2, −*y*+1, *z*.

### Hydrogen-bond geometry (Å, º)

**Table:**

*D*—H···*A*	*D*—H	H···*A*	*D*···*A*	*D*—H···*A*
O4—H41···O2^ii^	0.79 (4)	1.95 (3)	2.7367 (17)	171 (4)

Symmetry code: (ii) *x*, −*y*+1, *z*−1/2.
